# Spontaneous Resolution of Extensive Haemosiderin Skin Staining Following Iron Infusion During Pregnancy

**DOI:** 10.7759/cureus.105577

**Published:** 2026-03-21

**Authors:** Tina Barez, Bradley de Vries, Alice Burton

**Affiliations:** 1 Obstetrics and Gynaecology, Royal Prince Alfred Hospital, Sydney, AUS; 2 Obstetrics and Gynaecology, Royal North Shore Hospital, Sydney, AUS

**Keywords:** adverse outcome, cutaneous hyperpigmentation, haemosiderin staining, intravenous iron supplement, iron deficiency anaemia, iron extravasation, iron (iii) hydroxide polymaltose complex (ipc), quality of antenatal care, spontaneous resolution of lesions, treatment outcome

## Abstract

Intravenous iron is increasingly used in pregnancy for the treatment of iron deficiency anaemia due to its efficacy and rapid haematologic response. Cutaneous haemosiderin staining secondary to iron extravasation is a rare but distressing complication that is frequently described as persistent or permanent. Although therapeutic interventions have been reported and rare spontaneous cases may exist in the broader dermatologic literature, we found no well-documented reports demonstrating spontaneous improvement or complete resolution without treatment, particularly with long-term follow-up and photographic confirmation.

We present the case of a nulliparous woman in her 20s who developed extensive haemosiderin staining of the right upper arm following peripheral intravenous iron polymaltose infusion at 22 weeks of gestation. Discolouration progressed over one month and was associated with intermittent discomfort. Laser therapy was recommended by the dermatology team for cosmetic management; however, the patient elected for expectant management. No therapeutic intervention was undertaken. Gradual fading became apparent approximately nine months after the extravasation event, with complete resolution confirmed at 39 months during assessment in a subsequent pregnancy. Although the interval between these reviews was extended, the patient reported steady improvement without any interim treatment.

To our knowledge, this case provides the first clearly documented longitudinal evidence of complete spontaneous resolution of extensive iron staining, supported by long-term photographic follow-up. This conclusion is based on a literature search conducted via PubMed and MEDLINE, which revealed no previously reported cases of spontaneous resolution without intervention. Recognition that natural resolution may occur could support a shift toward expectant first-line management and offer reassurance, particularly for patients who may not have access to expensive dermatologic treatments.

## Introduction

Anaemia in pregnancy remains a critical global health concern, with iron deficiency representing the most common aetiology [1,2]. The World Health Organization reports 32 million (37%) pregnant women are affected by anaemia annually, with over 50% being attributed to iron deficiency [2]. Intravenous iron therapy has become increasingly utilised, with recent Australian data indicating that infusions are now administered to roughly one in seven pregnant women in some centres, reflecting rapid expansion due to improved tolerability and more rapid correction of haematologic indices compared with oral supplementation [3,4]. A survey of Australian and New Zealand obstetricians found that almost all prescribed intravenous iron in pregnancy, with more than half reporting at least occasional use as initial therapy [5].

Although generally safe, parenteral iron administration carries a rare risk of extravasation resulting in dermal haemosiderin deposition and visible hyperpigmentation, with clinical trials reporting haemosiderin staining rates of 0.68-1.3% [6,7]. This complication results from the leakage of intravenous iron into the dermis and subcutaneous tissue, followed by macrophage uptake and deposition as haemosiderin, producing brown to slate‑grey hyperpigmentation. Reports of haemosiderin staining have increased in parallel with the broader use of intravenous iron formulations [5,8]. The complication is widely described as persistent and potentially permanent, with many reports focusing on cosmetic distress and referral for laser therapy [8].

The natural history of iron staining remains poorly defined. To characterise existing evidence, we conducted a literature search of PubMed and MEDLINE over the past 10 years using combinations of the terms "intravenous iron", "iron infusion", "extravasation", "haemosiderin staining", "skin staining", "hyperpigmentation", "spontaneous resolution", and "natural history". We identified case reports and series describing prolonged pigmentation and laser-based interventions but found no clearly documented cases demonstrating complete spontaneous resolution without treatment and with long-term photographic evidence [8]. This absence of documented natural recovery may inadvertently bias management toward early procedural intervention. We present a case of extensive haemosiderin staining during pregnancy that resolved completely without intervention over more than three years of follow-up, providing longitudinal evidence that natural resolution can occur and supporting the consideration of expectant first-line management in selected patients.

## Case presentation

A nulliparous woman in her 20s, residing in Sydney, Australia, with Fitzpatrick skin type II, was diagnosed with symptomatic iron deficiency during her first pregnancy. At 22 weeks of gestation, laboratory investigations demonstrated haemoglobin at 117 g/L, ferritin at 14 µg/L, and transferrin saturation at 14%, and she reported fatigue. She had previously received oral iron supplementation with these indices and symptoms showing inadequate improvement.

**Figure 1:**
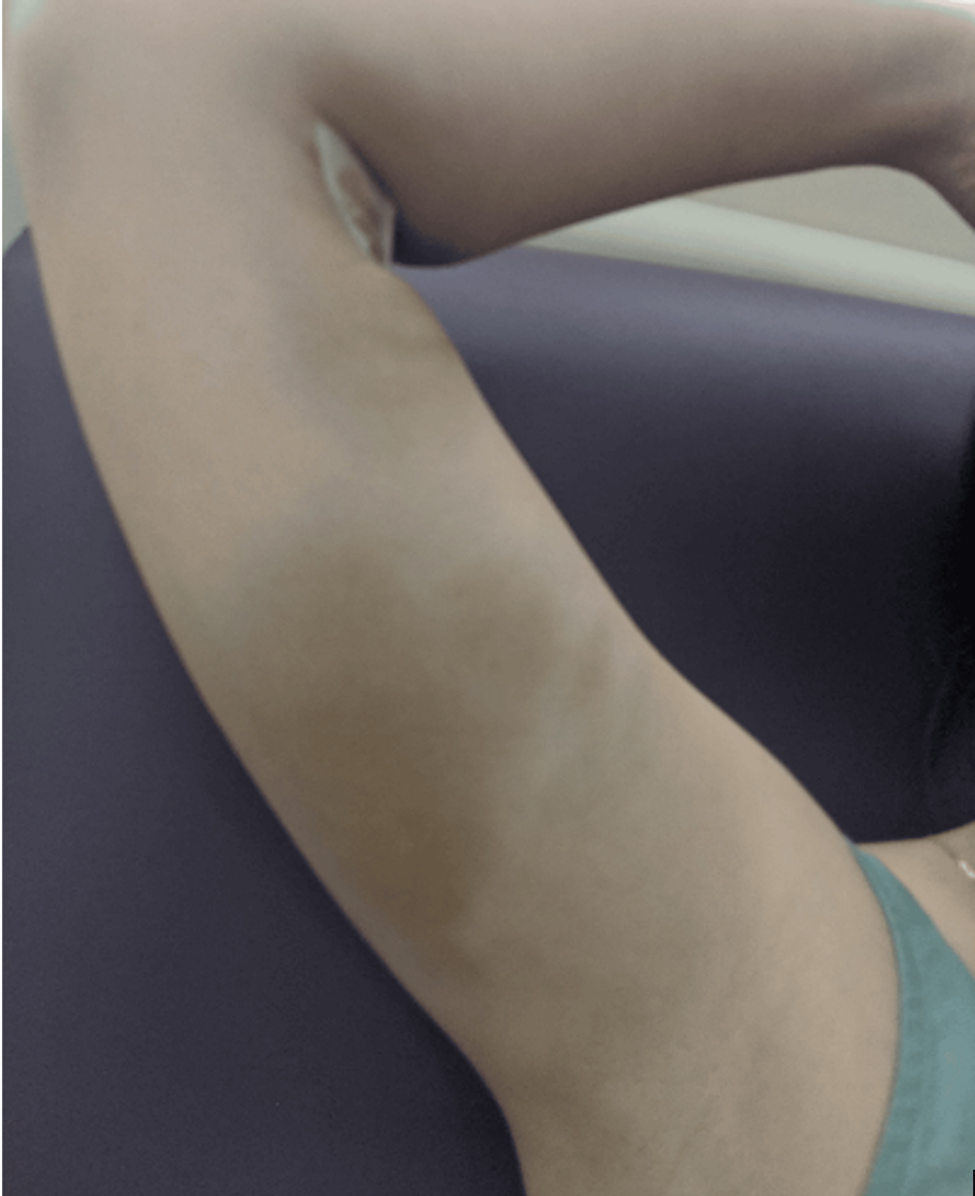
Four hours after intravenous iron infusion Clinical photograph taken four hours after peripheral intravenous iron polymaltose infusion via a cannula in the right cubital fossa. Swelling and ecchymosis with tan-brown discolouration are visible extending from the cubital fossa proximally along the medial upper arm toward the axilla, consistent with acute iron extravasation, with focal areas measuring approximately 5×3 cm at the cubital fossa and 5×5 cm in the proximal medial upper arm.

After informed consent, intravenous iron polymaltose 1000 mg diluted in 250 mL of 0.9% sodium chloride was administered via peripheral venous cannula in the right cubital fossa according to local protocol. The infusion was commenced at 40 mL/hr for 15 minutes and, in the absence of adverse symptoms, increased to 250 mL/hr for a total duration of approximately 75 minutes. Four hours following infusion, the patient developed swelling and a poorly demarcated tan-brown patch of cutaneous discolouration with an ecchymotic component extending from the cubital fossa proximally along the medial upper arm (Figure 1). At this time, the most intense staining measured approximately 5×3 cm at the cubital fossa, with a further area of tan-brown discolouration of approximately 5×5 cm in the proximal medial upper arm beneath the axilla. The presumptive diagnosis was haemosiderin staining secondary to iron extravasation. Plastic Surgery and Dermatology teams confirmed the diagnosis. Postpartum laser therapy was discussed as per dermatological suggestion, with counselling regarding cost, multiple sessions, and variable efficacy.

**Figure 2:**
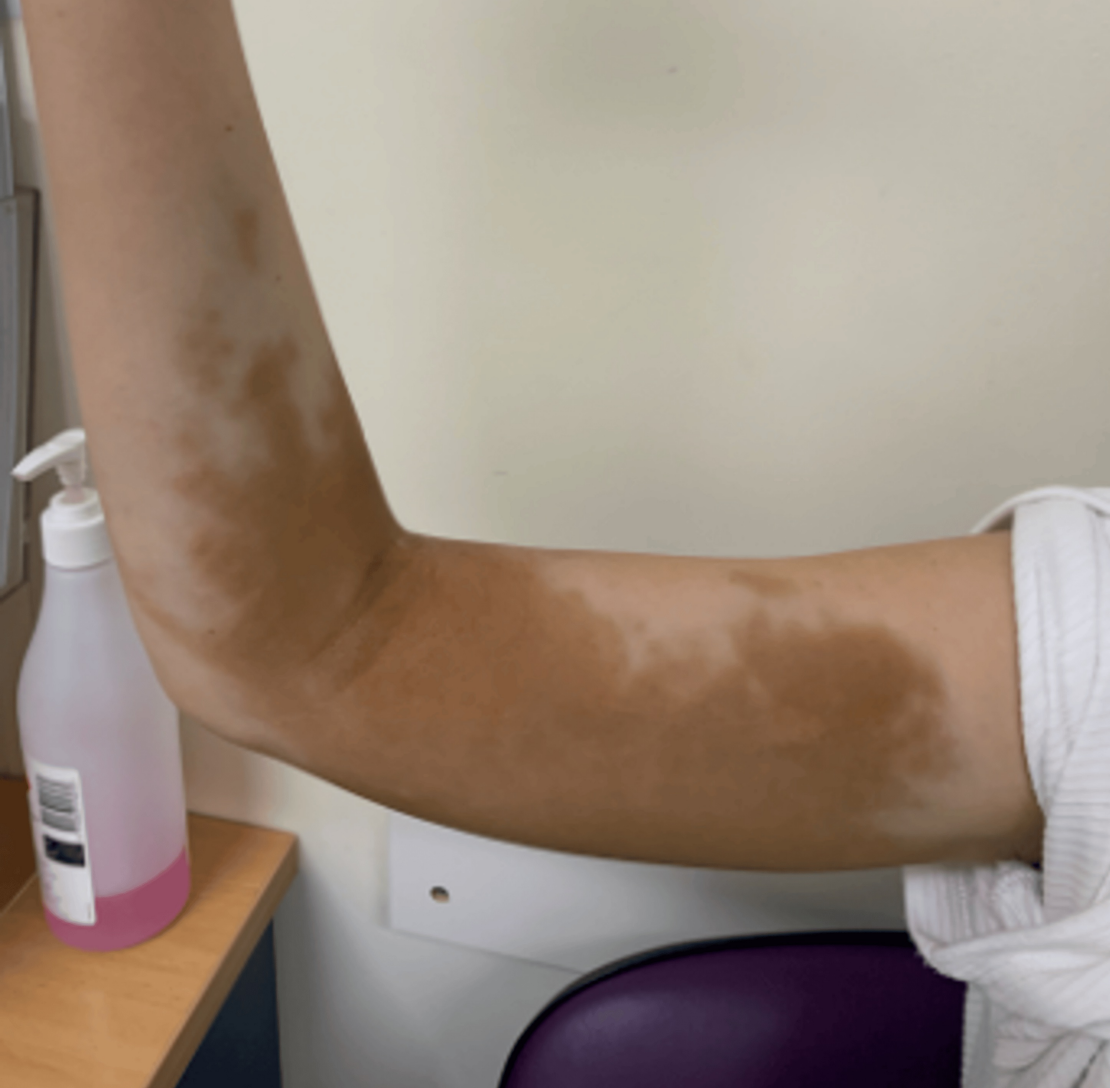
One month after intravenous iron extravasation Clinical photograph demonstrating the progression of cutaneous hyperpigmentation one month after the infusion event. Brown discolouration extends across the cubital fossa, distally to the proximal third of the forearm, and proximally along the upper arm to the axilla, involving the entire posterior aspect of the arm, with an estimated maximal area of involvement of approximately 15×7 cm.

Over the subsequent month, discolouration extended to involve the cubital fossa and posteromedial aspect of the arm (Figure 2), with an estimated area of involvement of approximately 15×7 cm spanning the medial and posterior upper arm. The patient experienced mild, self‑limiting aching in the affected area that did not affect function or influence management decisions. Her primary concern remained cosmetic appearance.

**Figure 3:**
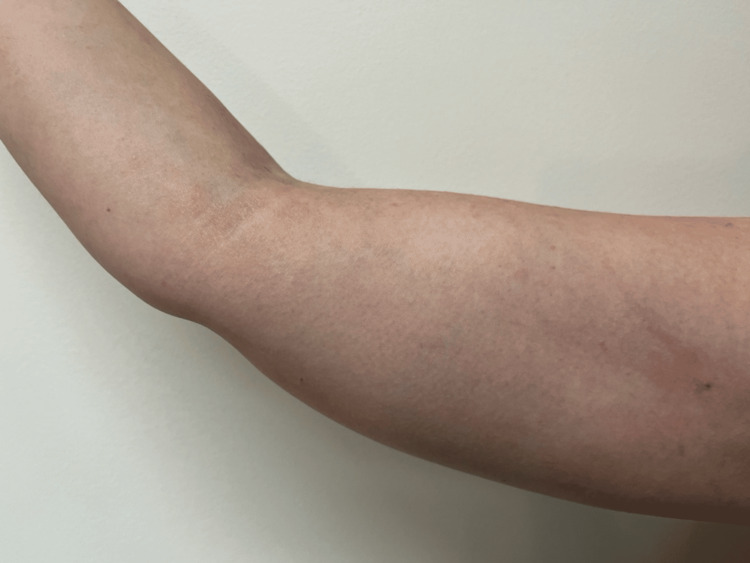
39 months post-extravasation Clinical photograph obtained 39 months after the initial event demonstrating the complete spontaneous resolution of the previously documented haemosiderin staining, with normal skin pigmentation and no visible residual discolouration.

The patient ultimately elected for expectant management. At the routine postpartum follow‑up, haemoglobin had improved to 129 g/L, with ferritin at 39 µg/L and serum iron at 16 µmol/L, consistent with the correction of iron deficiency. She was reviewed in a subsequent pregnancy three years later, at which point the skin staining was not detectable. Based on patient recall, gradual fading had begun approximately nine months after the extravasation event. At 39 months post-event, complete resolution of pigmentation was confirmed, with no residual discolouration (Figure 3).

A brief timeline of the clinical course is provided in Table 1.

**Table 1:** Timeline of the clinical course A brief timeline of the clinical course is provided to summarise key events from infusion to resolution.

Time from infusion	Gestation/context	Clinical findings/events
Day 0	22 weeks' gestation	Intravenous iron polymaltose infusion completed
+4 hours	22 weeks' gestation	Swelling and tan-brown discolouration of the medial upper arm; diagnosis of iron extravasation considered; specialist review and laser therapy discussed
1 month	Ongoing pregnancy	Progression to extensive patchy tan-brown macular hyperpigmentation involving the medial and posterior upper arm; mild intermittent aching discomfort
~9 months	Postpartum	Patient reports the onset of gradual fading of pigmentation
39 months	Subsequent pregnancy review	Complete clinical resolution of staining; no residual discolouration

## Discussion

Iron deficiency anaemia remains a condition of significant global morbidity, particularly among pregnant women. Untreated maternal iron deficiency anaemia has been associated with adverse obstetric and neonatal outcomes, including preterm birth and low birth weight, with potential long-term consequences for infant health [9]. Maternal complications may include increased susceptibility to peripartum and postpartum haemorrhage and a higher likelihood of requiring blood transfusion. Given these risks, timely identification and effective correction of iron deficiency anaemia are essential. Intravenous iron preparations, including ferric carboxymaltose and iron polymaltose, provide the rapid replenishment of iron stores and are widely used when oral therapy is ineffective or poorly tolerated [9]. Recent literature suggests that intravenous iron administered in the second and third trimesters is safe and achieves faster haematologic improvement with fewer gastrointestinal adverse effects than with oral iron [9]. Consequently, the use of parenteral iron continues to expand in obstetric and general medical practice [3,5].

A recognised risk of intravenous iron therapy is cutaneous staining, occurring in up to 1% of infusions [9,10]. Cutaneous haemosiderin staining results from the extravasation of intravenous iron into the dermis and subcutaneous tissue, followed by macrophage uptake and deposition of haemosiderin, producing brown to slate-grey hyperpigmentation [8].

The natural history of haemosiderin skin staining is not well-described. The largest published series of skin staining after iron infusion is a retrospective analysis of the French national pharmacovigilance database by Hermitte-Gandoliere et al. [11]. This study identified 51 cases of cutaneous pigmentation related to intravenous iron extravasation between 2000 and 2016. Of these, pigmentation was reported to persist beyond one month in 19 patients (37.2%) and beyond six months in nine patients (17.6%), although the completeness and duration of follow-up varied. Resolution was explicitly documented in only three cases, and it is unclear whether the remaining patients experienced improvement, underwent treatment (such as laser therapy), or were simply not followed longitudinally. Importantly, management strategies were not systematically described, and procedural details regarding any interventions were largely absent.

Individual case reports similarly describe prolonged pigmentation lasting months to years, frequently prompting referral for laser therapy [12,13].

Available treatment options for cutaneous haemosiderin staining remain limited and are not guided by formal consensus recommendations. Conservative approaches such as topical depigmenting agents, massage, and lymphatic drainage have been attempted but have not demonstrated consistent efficacy [14]. Current interventional management primarily involves laser therapy, particularly Q-switched systems including 532 nm and 1064 nm neodymium-doped yttrium aluminum garnet (Nd:YAG) lasers [14]. Reported case series suggest that multiple sessions, often four to six treatments over many months, may produce partial to substantial fading, and in some instances complete clearance, though results are variable [14,15]. Treatment response appears influenced by lesion depth, chronicity, and individual skin characteristics. Importantly, complete clearance is not guaranteed, and recurrence has not been systematically studied. Financial costs may be considerable, and treatment is frequently not publicly funded. Access to dermatologic laser services may be limited in rural or resource-constrained settings.

The current case demonstrates that spontaneous resolution of haemosiderin skin staining is possible. Expectant management may represent a reasonable first-line approach, with the understanding that improvement may take months to years. This report has several strengths, including prolonged longitudinal follow-up exceeding three years, serial photographic documentation at defined time points, and multidisciplinary input from Plastic Surgery and Dermatology supporting the diagnosis of haemosiderin staining secondary to iron extravasation. It also contributes novel evidence regarding the potential for complete spontaneous resolution of extensive staining, addressing an acknowledged gap in the literature, which predominantly emphasises persistent pigmentation and early procedural intervention.

However, important limitations must be recognised. As a single-patient observational case, the findings are not generalisable and cannot predict outcomes for all patients with iron extravasation. Objective pigment quantification was not performed, and assessment of improvement relied on clinical photographs, approximate linear measurements, and patient report, with the reported onset of fading at around nine months based on recall rather than protocolised assessment. Follow-up was opportunistic rather than protocol-driven, and images were obtained without scale markers and under non-standardised conditions, limiting precise comparison of lesion size over time. Finally, resolution was defined clinically and photographically; no dermoscopy, reflectance confocal microscopy, or histological assessment was undertaken, so subclinical residual dermal iron deposition cannot be excluded. These factors should be considered when extrapolating the implications of this case to broader clinical practice.

Prevention remains important. Risk factors for extravasation include fragile peripheral veins, suboptimal cannula placement, infusion under pressure, and failure to discontinue infusion when early symptoms such as pain or burning occur. Careful vein selection, secure cannula fixation, slow infusion rates, and vigilant monitoring are recommended.

## Conclusions

Haemosiderin staining following intravenous iron extravasation is a rare but distressing complication frequently described as persistent or permanent. This case illustrates complete spontaneous resolution with conservative management and, to our knowledge, adds further longitudinal evidence that such resolution is possible in extensive staining.

Recognition that natural resolution may occur could help inform clinical counselling, support the consideration of expectant first-line care in selected patients, and reduce unnecessary financial burden, particularly for those with limited access to dermatologic procedures.
